# Berberine ameliorates aGVHD by gut microbiota remodelling, TLR4 signalling suppression and colonic barrier repairment for NLRP3 inflammasome inhibition

**DOI:** 10.1111/jcmm.17158

**Published:** 2022-01-05

**Authors:** Yanna Zhao, Jiefeng Huang, Tianyi Li, Shuijuan Zhang, Chengping Wen, Lipei Wang

**Affiliations:** ^1^ College of Basic Medical Science Zhejiang Chinese Medical University Hangzhou China; ^2^ Institute of Hematology Research The First Affiliated Hospital of Zhejiang Chinese Medical University Hangzhou China

**Keywords:** acute graft‐versus‐host disease, berberine, cytokines, gut microbiota, NLRP3 inflammasome, TLR4 signaling pathway

## Abstract

Berberine (BBR), an isoquinoline alkaloid, is used to treat gastrointestinal disorders as an herbal medicine in China. The aim of this study was to investigate the anti‐inflammatory activities of BBR in a mouse model with acute graft‐versus‐host disease (aGVHD). Mice were intravenously injected with bone marrow cells from donors combined with splenocytes to develop aGVHD. The body weight, survival rate and clinical scores were monitored. Then the levels of inflammatory cytokines, histological changes (lung, liver and colon), colonic mucosal barrier and gut microbiota were analysed. Moreover, the toll‐like receptor 4 (TLR4)/myeloid differentiation primary response gene 88 (Myd88)/nuclear factor‐κB signalling pathway, NLRP3 inflammasome and its cytokines’ expressions were determined. The results showed that the gavage of BBR lessened GVHD‐induced weight loss, high mortality and clinical scores, inhibited inflammation and target organs damages and prevented GVHD‐indued colonic barrier damage. Additionally, BBR modulated gut microbiota, suppressed the activation of the TLR4 signaling pathway and inhibited NLRP3 inflammasome and its cytokine release. This study indicated that BBR might be a potential therapy for aGVHD through NLRP3 inflammasome inhibition.

## 1 INTRODUCTION

Allogeneic haematopoietic stem cell transplantation (allo‐HSCT) is effective for many haematologic and hereditary diseases as a treatment strategy.^1^ During allo‐HSCT, donor‐derived immunocompetent cells may cause the organs damage in recipient, which is known as GVHD. The major cause of non‐relapse mortality is acute GVHD (aGVHD).^2^ The pathophysiology of aGVHD involves the dysregulation of inflammatory cytokine cascade.^3^

Studies have confirmed the relationship between gut microbiota and the development of aGVHD.^4^ Gut microbiota plays extensive roles containing immunological functions. Gut microbiota can interact with the immune components of microbial immunity, although it is a non‐immune component. The microbiota composition dysregulation or alterations would be resulted in the pathogenesis of GVHD. Before allo‐HSCT, recipients undergo a conditioning regimen, which induces damage in tissue, allowing translocation of bacterial products from mucosa into the internal milieu where a “cytokine storm” is provoked.^5^ However, the molecular mechanism inducing cytokine production is still poorly understood.

Increasing studies have suggested that NLRP3 inflammasome plays a key role in the shaping of gut microbiota composition. NLRP3 inflammasome, as the essential step for the activation of caspase‐1, controls the activation of inflammatory cytokines.^6^ The NLRP3 inflammasome activation causes the active form of the precursor protein pro‐IL‐1β by cleavage, and the release of inflammatory factors, such as TNF‐α, IFN‐γ and IL‐1, can induce “cytokine storm” and altered gut microbiota composition, which causes vicious spiral. The clinical use of immunosuppressive agents, cytotoxic drugs, or in vitro/in vivo T‐depletion are used in aGVHD, but it also results in a high relapse rate after transplantation.^7^ Hence, other valid strategy for the relieve of GVHD‐induced damages is necessary, NLRP3 would be the therapeutic target of GVHD.

Berberine (BBR) is extracted from the Chinese herb Coptis chinensis (Huang‐Lian, a traditional Chinese herb in medicine).^8^ As the major pharmacological component of Huanglian, BBR and its containing herbs are used to treat bacterial diarrhoea and other intestinal infections in China for thousands of years. Recently, some researchers found that BBR was clinically effective in type 2 diabetes treatment and significantly total cholesterol and low‐density lipoprotein cholesterol levels decreasing.^9^ However, the mode of action of BBR remains a paradox due to its absorbed poorly into the blood from the intestine, gut microbiota modulation is the most likely the mechanisms of its pharmacological effect,^10^ similarly to many other traditional Chinese herbal medicines.^11^

In this study, we aimed to explore whether BBR had any influences on inflammatory cytokines, the pathological changes of target organs, especially gut microbiota and NLRP3 inflammasome in mice with aGVHD. Notably, this study was conductive to elucidate the pharmacological mechanisms of BBR and suggested that BBR may be a possible clinical gut microbiota modulator for the treatment of aGVHD.

## 2 MATERIALS AND METHODS

### 2.1 Animals

Female BALB/c and male C57BL/6 mice (5–6 weeks, weight 20–22 g), were purchased from Shanghai SLAC Experimental Animals (China) and were all maintained in specific pathogen free conditions. The experiments were performed in the facility of Zhejiang Chinese Medical University and mice were cared for according to the guidelines of the National Science and Technology Committee of China.

### 2.2 Bone marrow transplantation and induction of acute GvHD

Donor mice were sacrificed via cervical dislocation under anaesthetization. Then, flushed femurs and tibias for the preparation of bone marrow cells (BMCs) with Roswell Park Memorial Institute medium 1640 and Dulbecco's Phosphate Buffered Saline (DPBS, D8662, Sigma‐Aldrich). Splenocytes were isolated by a cell strainer from the spleen tissue. In GvHD induction process, after the adjustment of determined optimal dose, 2 × 10^7^ BMCs combined with 2 × 10^7^ splenocytes that both from either allogeneic (C57BL/6) donors were injected intravenously into the recipients (BALB/c) which treated with 9 Gy TBI, at day 0.

### 2.3 Experimental design

BALB/c mice were divided into three groups randomly: Control Group (normal mice were provided normal food and water without any treatment), Model Group (aGVHD mice were not administered any treatment), Berberine (CAS: 633‐65‐8, Purity: over 98%, Sub‐formula: C20H18CLNO4·2H2O, Sub‐quantity: 336) Group (consisting of aGVHD mice treated by BBR (50 mg/kg/day) after transplantation).

### 2.4 aGVHD monitoring and scoring

The body weight and clinical symptoms were recorded every 4 days, and the survival was monitored every day. A clinical scoring system, described previously, was applied to evaluate the severity of GVHD, including weight loss, activity, posture (hunching), fur texture and skin integrity.^12^ Experimental mice, placed in coded cages, were assessed once a week. Correspondingly, scores range from 0 to 2 were used to record the gradational changes which according to each criterion, and then created the clinical index through the sum of 5 criteria scores above (maximum, 10).

### 2.5 Cytokine measurements

Serum samples were collected from whole blood without anticoagulants and centrifuged at 4℃/1000 g for 10 min to collect the serum supernatant for analysis. Then, the level of murine TNF‐α, IL‐1β, IFN‐γ, MCP‐1, IL‐6, IL‐18 (CUSABIO) and LBP (Abcam, ab269542) were assayed quantitatively via ELISA kits (CUSABIO) between day 4 and 6 after bone marrow transplantation (BMT), following the manufacture protocols. Next, we analysed microwell strips by using a microwell reader (Molecular Devices), weighed the extracted colon tissues, homogenized tissues in the tissue extraction reagent for 3 min on ice and acquired the supernatants after 4℃/10,000 *g* centrifugation for 5 min, then, stored at −20℃. Finally, we detected the levels of IL‐1β, TNF‐α and IL‐6 in colon homogenates through ELISA assay.

### 2.6 Histology

Mice were sacrificed to harvest aGVHD target organs (lung, liver, colon) from recipients on day 25, then immediately fixed with 4% formalin and embedded into paraffin following standard procedures. Afterwards, we mounted the sections (5 μm) on the slide for H&E staining and estimated histologic changes of liver and small intestine by using the semiquantitative scoring system (SSS) mentioned above.^13^ Interstitial/alveolar inflammation and periluminal bronchial/vascular infiltration are histological changes compatible with GVHD in the lungs. Grading categories of interstitial/alveolar inflammation: none = 0, mild = 1 (<25%), moderate = 2 (25%–50%) and severe =3 (>50%). Liver pathology severity was evaluated with GVHD grading system recomposed from Cooke,^14^ which includes portal infiltrates, cell apoptosis, vascular endothelialitis, bile duct injury and lobular infiltrates (normal = 0, mild = 1, moderate = 2 and severe = 3). the histological changes in colon also evaluated by SSS as well. Changes of compatible with GVHD was defined by lamina propria inflammation, goblet cell depletion and ulceration of the colonic mucosa. All slides were coded and a Nikon Eclipse E400 microscope was utilized for visualization. Ultimately, a Zeiss Axiom camera was applied for image acquisition and analysed with software AxioVision 3.0.6 SPZ (Zeiss).

### 2.7 Immunohistochemistry

Paraffin embedded colon tissue sections were dewaxed, re‐hydrated and antigen retrieval. Incubated the sections in hydrogen peroxide (3%) and normal goat serum (Solarbio) for 15 min respectively. Then, the sections were incubated overnight with the primary antibody, Occludin antibody (eBioscince) and ZO1 antibody (eBioscince) in sequence (1:200 dilution), at 4℃. Afterwards, HRP‐labeled goat anti‐rabbit IgG (eBioscince) as the secondary antibody (1:500 dilution) to incubate with the sections for 60 min at 4℃. All the sections were then stained with diaminobenzidine (Solarbio) and counter‐stained with hematoxylin. Later, observation was made under microscope (200×, OLYMPUS) and the quantitative analysis was performed by Image‐pro Plus software (Media Cybernetics).

### 2.8 16S rRNA gene tag sequencing

Bacterial DNA was extracted by QIAmp DNA^®^ stool kit (Qiagen). Bacterial species were identified and classified based on 16S rRNA gene sequencing.^15^, ^16^ Briefly, samples were centrifuged at 20,000 *g* for 30 min at 4℃ to extract the total genomic DNA. The integrity, concentration and quality of the total DNA were determined by agarose gel electrophoresis through A260 and A260 to A280 ratio. After extraction, pooled and individual DNA samples were quantified by QuantiGene 2.0 Reagent System (Panomics/Affymetrix), following protocols.

Bacterial DNA was extracted with QIAmp DNA^®^ stool kit (Qiagen) and were determined by agarose gel electrophoresis (1% w/v agarose). Additionally, NanoDrop2000 spectrophotometer (Thermo Scientific) was used to quantify the DNA and stored at −20℃ for sequencing on the Illumina MiSeq platform. Next, we applied the primers 319f (5′‐ACTCCTACGGGAGGCAGCAG‐3′) and 806r (5′‐GGACTACHVGGGTWTCTAAT‐3′) to amplify the V3‐V4 region of ^16^S rRNA genes from duplicate DNA extractions. PCR amplifications were carried out in a 30 ml mixture that included 0.5 ml DMSO, 1.0 ml forward primer (10 mM), 1.0 ml reverse primer (10 mM), 5.0 ml DNA sample, 7.5 ml ddH_2_O and 15.0 ml of PCR Master Mix. Reaction conditions were 98℃ × 30 s, 30 cycles of (98℃ ×  15 s; 58℃ ×  15 s; 72℃ ×  15 s); and final extension 72℃  × 1 min. Then, PCR products were purified with the agarose gel DNA purification kit (Qiagen) and a TruSeqTM DNA sample preparation kit (Illumina Inc) was employed for the preparation of amplicon library. Sequencing reactions were performed by Illumina MiSeq sequencing (2 × 300 bp; Hangzhou Guhe Information and Technology Co., Ltd.).

### 2.9 Western Blot analysis

After extraction of total protein with RIPA and PMSF (Beyotime), protein concentrations were measured by BCA protein assay kit (Beyotime). Then, β‐actin was employed as an internal control and samples were separated on SDS‐PAGE gel and transferred to a nitrocellulose blotting membranes (A10464264, GE Healthcare Life Science). The membrane was incubated overnight at 4℃ with the primary antibody after blocked with BSA. Following, incubated the membranes with the appropriate secondary antibody for 1 h. Finally, the gray values of the protein bands were analysed by Gel‐Pro‐Analyzer software. Primary antibodies utilized in this experiments were as follows: Claudin‐1 (1:1000 dilution; Santa‐Cruz#166338), Claudin‐2 (1:1000 dilution; Santa‐Cruz#293233), NLRP3 antibody (1:500 dilution; Abcam#ab214185), ASC antibody (1:1000 dilution; Cell Signaling Technology#67824) and Caspase‐1 antibody (1:200 dilution; Abcam#ab1872), anti‐β‐actin (mouse, 1:1000, 3700, CST).

### 2.10 Real time‐PCR analysis

We extracted total RNA from colonic tissues by using Trizol reagent (Invitrogen) according to the standard protocol and then was reversely transcribed into cDNA. qRT‐PCR was assessed for the quantification of mRNA purity and concentrations via PowerUp SYBR Green Master Mix (Thermo Fisher Scientific) and 7500 Fast Real‐Time OCR System (Thermo Fisher Scientific). The sequences of primers were: TLR4 (Sense: 5′‐ATGGCATGGCTTACACCACC‐3′; Anti‐sense: 5′‐GAGGCCAATTTTGTCTCCACA‐3′), MyD88 (Sense: 5′‐TCATGTTCTCCATACCCTTGGT‐3′; Anti‐sense: 5′‐AAACTGCGAGTGGGGTCAG), NF‐κB p65 (Sense: 5′‐TGCGATTCCGCTATAAATGCG‐3′; Anti‐sense: 5′‐ACAAGTTCATGTGGATGAGGC‐3′), IL‐1β (Sense: 5′‐ACCTGTGTCTTTCCCGTGG‐3′; Anti‐sense: 5′‐TCATCTCGGAGCCTGTAGTG‐3′), IL‐18 (Sense: 5′‐CTGTTGGCCCAATTACTAACAG‐3′; Anti‐sense: 5′‐TCCCGAATTGGAAAGGGAAATA‐3′), IL‐6 (Sense: 5′‐CTCCCAACAGACCTGTCTATAC‐3′; Anti‐sense: 5′‐CCATTGCACAACTCTTTTCTCA‐3′) β‐actin (Sense: 5′‐CTACCGTCGTGACTTCGC‐3′; Anti‐sense: 5′‐GGGTGACATCTCCCTGTT‐3′).

### 2.11 Statistical analysis

Statistical analysis was performed by the SPSS 13.0 software. All data were presented as mean values ± standard deviation (mean ± SD). Statistical analysis of the original data was evaluated with the Student's *t*‐test. One‐Way ANOVA was used for single factor analysis of variance. When variance was uniform, S‐N‐K method was used for the comparison among groups. As for heterogeneity of variances, it would be compared with Dunnett's method. A *p* value of <0.05 was taken as statistically significant.

## 3 RESULTS

### 3.1 The administration of berberine significantly alleviates the severity of aGVHD

**FIGURE 1:**
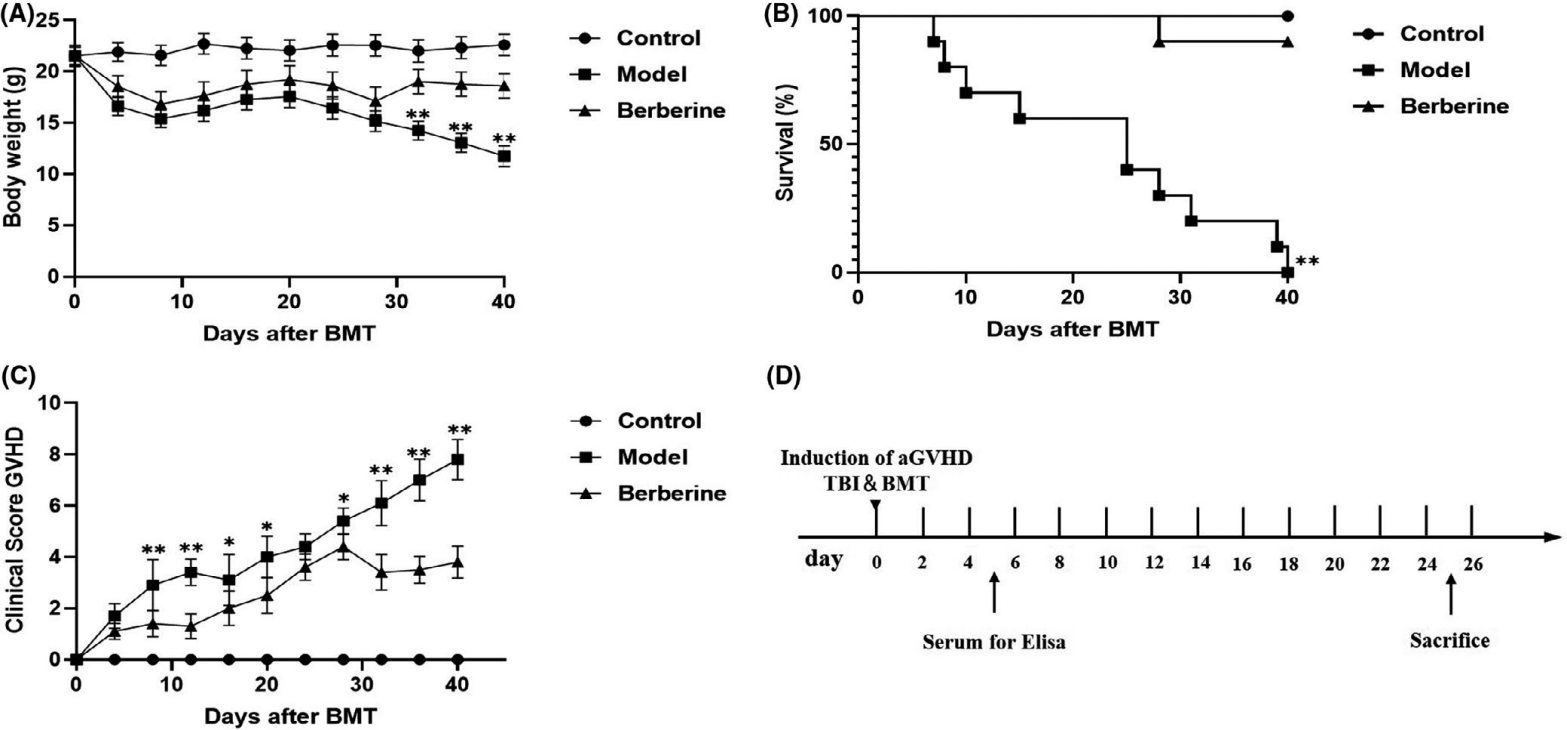
The mortality and morbidity of GVHD decreased in BMT by berberine (*N* = 10/group). (A) Changes in body weight every 4 days. (B) Percentage of survival. (C) Mean clinical scores every 4 days. (D) The flow chart of the following experimental procedures. Data are shown as mean ± SD and were analysed by Multiple *t* tests. *p < 0.05 and **p < 0.01 between model and berberine groups at the indicated time points. The graph (A and C) show the data combined with three independent experiments (10–15 animals in each group)

The administration of berberine prevented significant weight loss, while the model group body weight decreased until day 8, which may be the side‐effects of TBI. Then, the weight increased until day 28 and decreased once more. The mice body weight in berberine group kept higher than the model group with statistical significance at day 32 (*p* = 0.0075), day 36 (*p* = 0.0007) and day 40 (*p* < 0.0001) (Figure 1A). 100% of the mice died within 40 days after BMT, which is only 10% with treatment of berberine (Figure 1B). Clinical symptoms of GVHD were quantified by the scores summed for each of five parameters (0–2/each parameter): weight loss, posture, skin integrity, fur texture and activity, as previously reported.^17^ The higher scores mean the clinical symptoms are more severe. The scores of model group increased until day 12 and reduced until day 16, then the score displayed a stable increase. The rise in clinical score after day 28 was declined in berberine group. There were significant differences at day 8 (*p* = 0.0049), day 12 (*p* = 0.0057), day 16 (*p* = 0.0124), day 20 (*p* = 0.0117), day 28 (*p* = 0.0119), day 32 (*p* = 0.0012), day 36 (*p* = 0.0043) and day 40 (*p* = 0.0088) between two groups (Figure 1C).

### 3.2 Administration of berberine decreases proinflammatory cytokines in serum of aGVHD

**FIGURE 2:**
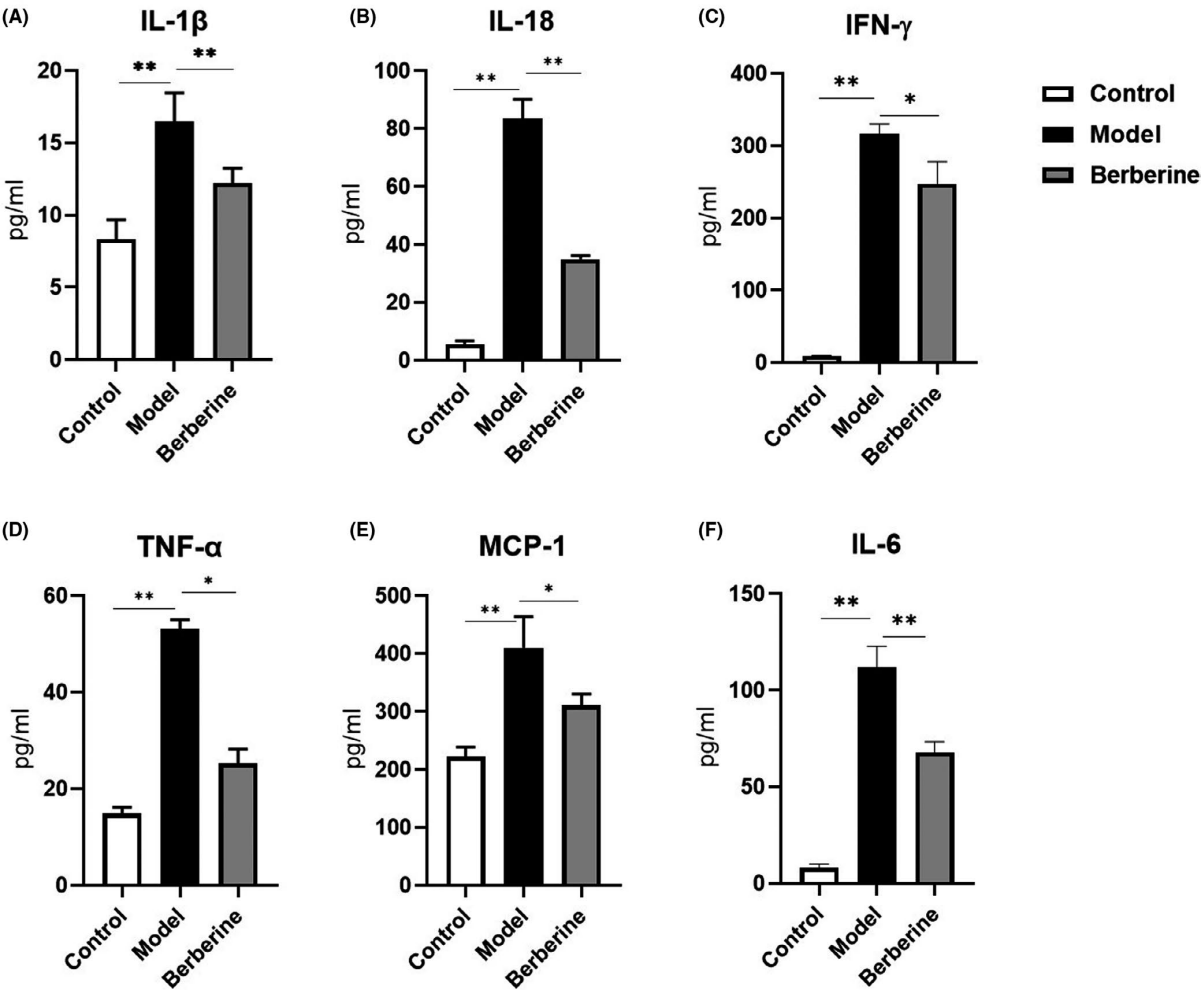
Effect of berberine on proinflammatory cytokines in serum (*N* = 5/group). IL‐1β (A), IL‐18 (B), IFN‐γ (C), TNF‐α (D), MCP‐1 (E) and IL‐6 (F). Data are shown as mean ± SD and were analysed by one‐way ANOVA. ^*^ *p* < 0.05; ^**^ *p* < 0.01

Some studies have demonstrated that proinflammatory cytokine release plays critical role in the development of aGVHD.^18^ We measured serum levels of IL‐1β, IL‐18, IFN‐γ, TNF‐α, MCP‐1 and IL‐6 between day 4 and 6 after BMT, a time point that indicates the break out of mortality by aGVHD. As shown in Figure 2, serum IL‐1β, IL‐18, IFN‐γ, TNF‐α, MCP‐1 and IL‐6 levels were markedly increased after BMT in aGVHD model compared with normal control, while decreased in berberine‐treated mice.

### 3.3 Berberine attenuates the pathological changes of target organs

**FIGURE 3:**
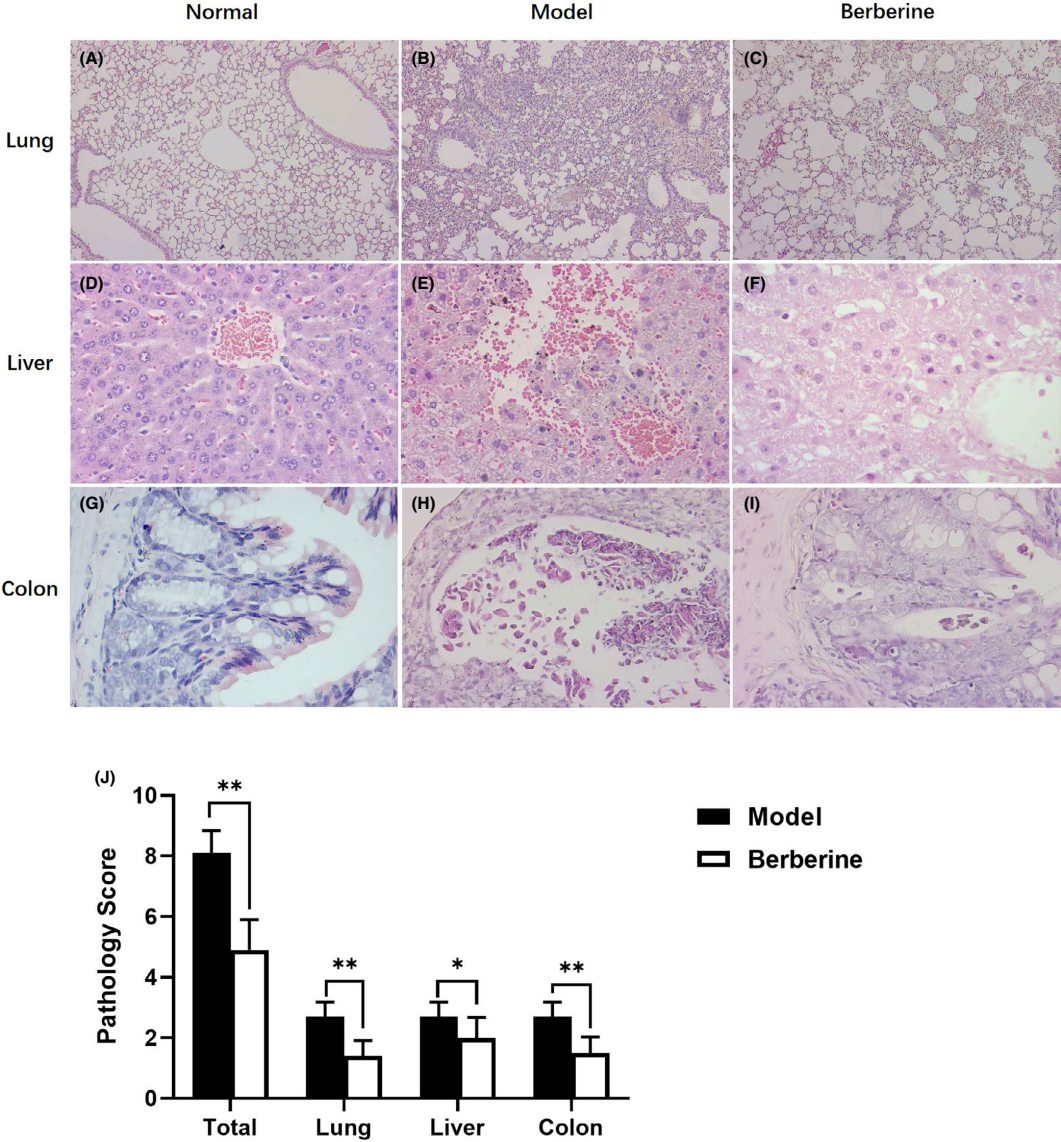
Attenuation of pathologic damage in GVHD target organs treated by berberine (*N* = 10/group). (A–C) Histology of lung in each group (100×). (D–F) Histology of liver in each group (400×). (G–I) Histology of colon in each group (400×). (J) The pathology score of the lung, liver and colon in three groups using SSS as detailed in Methods section. Data are shown as mean ± SD and were analysed by one‐way ANOVA. ^*^ *p* < 0.05 and ^**^ *p* < 0.01

We also examined whether any improvement of GVHD pathology is by berberine. Since the mice in model group died mostly from day 25 after BMT, the mice were sacrificed on that day. The target organs (lung, liver and colon) were prepared as tissue sections. As shown in Figure 3A–C, the inflammatory infiltration was observed in interstitial and perivascular of lung, the alveolar spaces were filled with the exudate in the model group; however, the inflammatory infiltration decreased in the berberine group. Figure 3D–F showed the structure of hepatic lobule was unclear with haemorrhage and various degrees of liver necrosis, especially near the central veins in model mice, when most of livers presented cellular swelling and the focal degeneration and necrosis were inconspicuous after berberine treatment. We further performed histopathological examination of the colon. The mucosal surface ulceration and lamina propria destruction were observed in model group, while colon in berberine group had normal appearing mucosa with less villous blunting, crypt destruction, inflammatory infiltration and preserved goblet cell content (Figure 3G–I). By the SSS as detailed in Methods section, the pathology score composed of histologic examination of target organs (lung, liver and colon) was significantly reduced in mice treated with berberine versus model group. It was clearly demonstrated that berberine ameliorated GVHD in lung, liver and colon with statistical significance (*p* = 0.0023), as shown in Figure 3J.

### 3.4 Berberine prevented GVHD‐induced colonic barrier dysfunction

**FIGURE 4:**
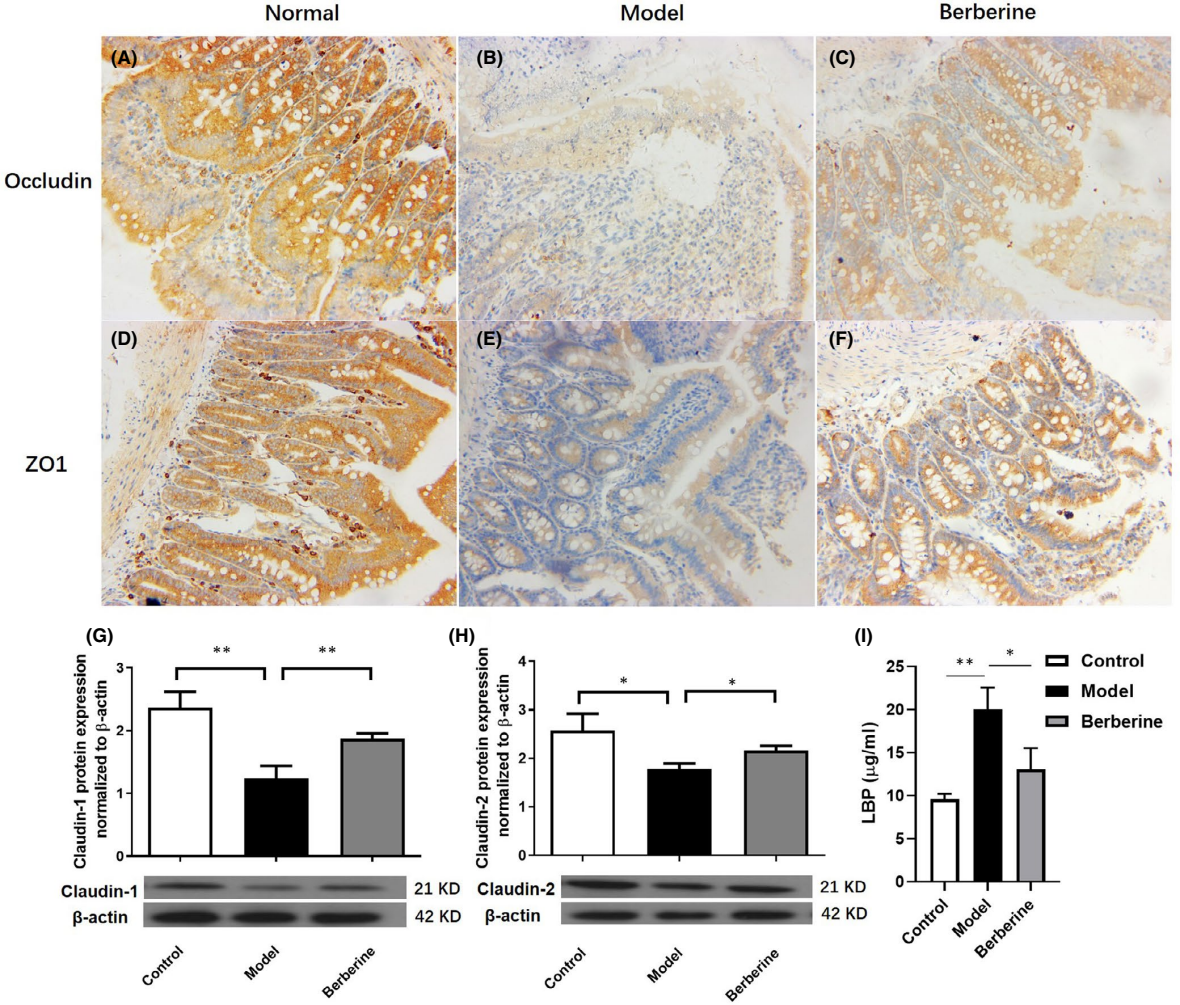
Berberine repaired the colonic barrier damage in GVHD mice. (A‐C) Expression of Occludin in each group (200×) and (D‐F) expression of ZO1 in each group (200×) (*N* = 10/group). Protein expression of Claudin‐1 (G) and Claudin‐2 (H) in colon tissue (*N* = 3/group). (I) Serum LBP concentration was determined by ELISA (*N* = 5/group). Data are shown as mean ± SD and were analysed by one‐way ANOVA. ^*^ *p* < 0.05; ^**^ *p* < 0.01

To evaluate the colonic tight junction function, the expression of Occludin and ZO1 was detected by IHC, while Claudin‐1 and Claudin‐2 by Western blot. The protein levels of Occludin, ZO1, Claudin‐1 and Claudin‐2 were lower in model colons in the comparison with the control mice (Figure 4A,B,D,E,G,H). Our results proved that aGVHD lowered the expression of tight junction proteins indicated the dysfunction of severe colonic tight junction barrier. Berberine worked as the NLRP3 inflammasomes inhibitor as previous study described. To further confirm how berberine functioned in aGVHD colon, the tight junction proteins expression was detected in the colons of aGVHD mice which was increased by berberine treatment compared to the model mice (Figure 4C,F,G,H). Results of IHC and Western blot revealed that the colonic tight junction proteins reduction in aGVHD mice could be reversed by berberine treatment.

LBP is a major transporter of proinflammatory lipopolysaccharides (LPS) in the plasma which is often used as an indicator of colonic barrier integrity. The plasma LBP concentration increased in aGVHD, indicating colonic barrier impairment. However, berberine group was comparable with the control values, suggesting that berberine prevented colonic barrier degradation (Figure 4I).

These data disclosed that berberine could prevent the colonic barrier impairment caused by aGVHD.

### 3.5 Berberine partly recovered the intestinal dysbiosis in GVHD mice

**FIGURE 5:**
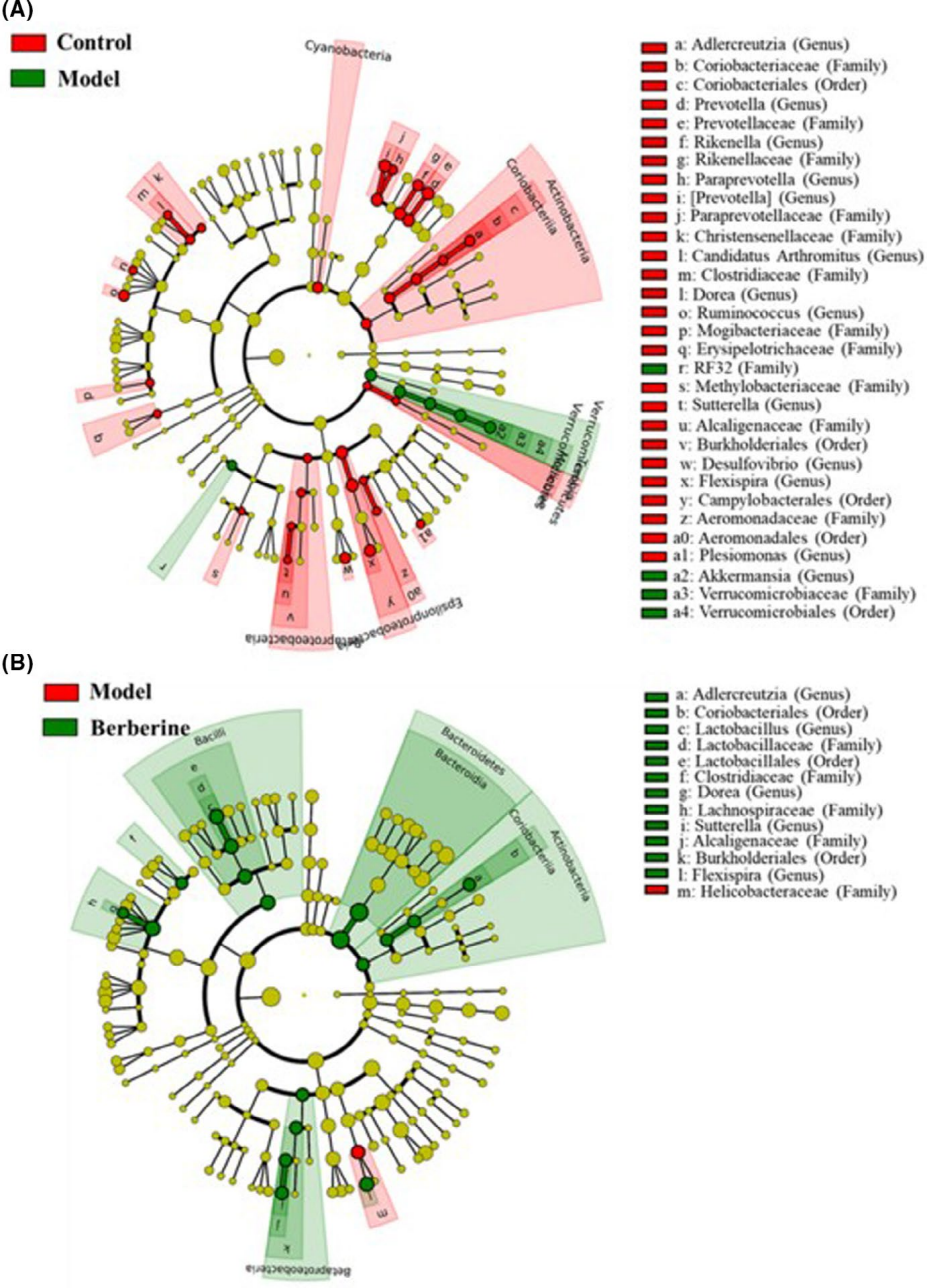
Berberine recovered the gut microbiome changes in GVHD Mice. Differentially expressed taxa with the LDA scores >2.0 and adjusted *p* values <0.05 between the control and model mice (A), and between the model mice and berberine treatment mice (B). Differences are represented by the colour of over‐represented taxa. Circles show phylogenetic levels from phylum (innermost layer) to genera (outermost layer)

As shown in Figure 5A, 40 bacterial taxa were significantly different between the control and model groups. At the level of phylum, the GVHD model mice had an increased abundance of Verrucomicrobia and the decrease abundance of Actinobacteria, Epsilonproteobacteria and Tenericutes. At the level of genus, 12 genera (*Adlercreutzia*, *Prevotella*, *Rikenella*, *Paraprevotella*, [*Prevotella*], *Candidatus Arthromitus*, *Dorea*, *Ruminococcus*, *Sutterella*, *Desulfovibrio*, *Flexispira* and *Plesiomonas*) were less abundant in the GVHD model mice, but only Akkermansia were more abundant in the GVHD model mice.

After berberine treatment, the gut microbiome was changed in GVHD model mice. As shown in Figure 5B, phylum Actinobacteria and Bacteroidetes and genus *Adlercreutzia*, *Lactobacillus*, *Dorea*, *Sutterella* and *Plesiomonas* were increased in the GVHD model mice after berberine treatment. Therefore, berberine could renovate the abundances of genus *Adlercreutzia*, *Dorea*, *Sutterella* and *Plesiomonas* and additionally increase the abundances of *Lactobacillus* in the treatment of GVHD mice.

### 3.6 Berberine reduced the expression of NLRP3 inflammasome and TLR4 signalling

**FIGURE 6:**
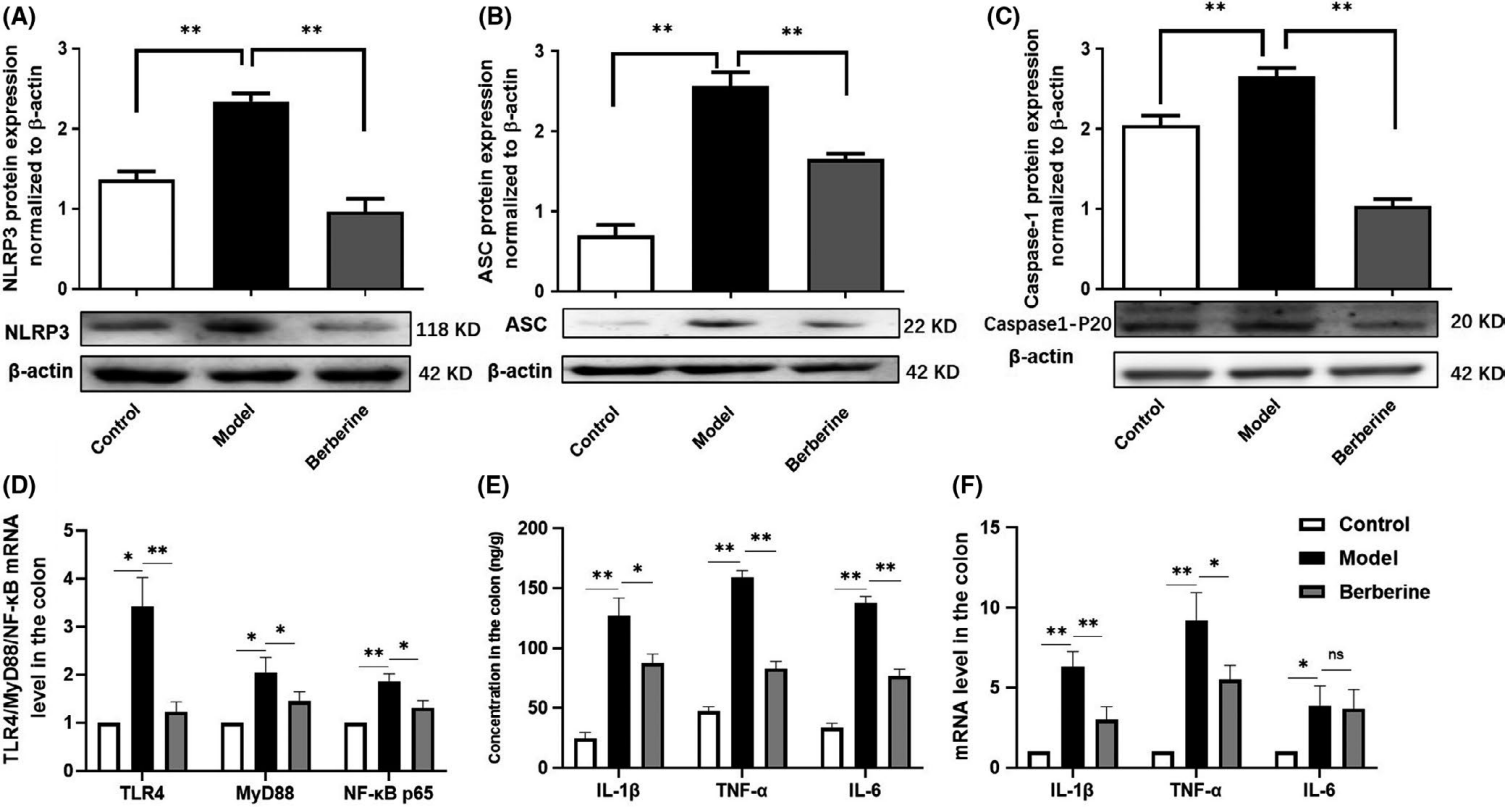
Suppression of gut NLRP3 inflammasome, TLR4 signalling and inflammatory cytokines in GVHD mice colonic tissues by berberine (*N* = 3/group). Protein expression of NLRP3 (A), ASC (B), and Caspase‐1 (C), and mRNA expression of TLR4/MyD88/NF‐κB (D) in colon tissue. The levels of inflammatory cytokines (IL‐1β, TNF‐α and IL‐6) from the colon tissue of GVHD mice were determined through ELISA (E) and Q‐PCR (F), respectively. We used the β‐actin as an internal control. Data are shown as mean ± SD. ^*^ *p* < 0.05; ^**^ *p* < 0.01; ns represents not significant (Figure A–C were analysed using one‐way ANOVA, Figure D–F were analysed by Multiple *t* tests)

We collected colon samples (*N* = 3/group) to detect the expression of the NLRP3 inflammasome, TLR4 signalling and its inflammatory cytokines. In the colon, NLRP3 inflammasome was activated and its inflammatory cytokines were released in the mice model of GVHD, while berberine could inhibit the NLRP3 inflammasome (Figure 6A–C), TLR4 signalling (Figure 6D) and suppressed inflammatory cytokines except IL‐6 (Figure 6E,F).

## 4 DISCUSSION

Graft‐versus‐host disease, as a main complication of allo‐HSCT, usually causes in severe injuries of organs and the significant mortality. Although some strategies are applied in the GVHD therapy, it remains the major cause of mortality which approaches nearly 15% of patients after allo‐HSCT.^19^

Berberine was proven to be safe for human and used for treating type 2 diabetes, NAFLD (non‐alcoholic fatty liver disease) and gastrointestinal disorders.^20^ It has been reported that BBR, be used alone or in combination with other compound, contributes to the attenuation of GVHD in mice,^21^ but its mechanism is still unclear. Previous studies had proved that BBR could alleviate colitis by suppressing the release of proinflammatory cytokines.^22^

Inflammatory cytokines are considered to be important in the pathogenesis of aGVHD.^23^ The strong release of proinflammatory cytokines such as TNF‐α, MCP‐1 and IFN‐γ is often characterized in the transplantation model, which contributed to target organ injury in GVHD significantly.^24^ As we all know, the gut microbiota plays a crucial part in the release of proinflammatory cytokines in GVHD. Before allo‐HSCT, recipients experience the conditioning regimen, which includes cytotoxic drugs and γ‐irradiation. Above regimen could induce the damages in tissue, bringing bacterial products to translocate from the intestinal mucosa into the lamina propria, where “cytokine storm” is provoked. Then “cytokine storm” induces the inflammation in the host, activation of the recipient's antigen‐presenting cells, and a followed allogeneic reaction mediated by donor T cells, which results in the cytokine response amplification.^5^ In this study, BBR was observed to markedly downregulate the expression of proinflammatory cytokines in the GVHD mice; however, the mechanism of cytokines suppression by BBR was unclear. In view of this, we hypothesized that BBR probably suppress GVHD‐induced inflammatory cytokines release via remodelling gut microbiota.

Berberine was used for the therapy of gastroenteritis, diarrhoea and other infectious diseases clinically for a long time. It is believed that BBR has antimicrobial effects on bacterial pathogens which is associated with its pesticide effect.^25^ The gut is a primary target organ and plays a major role in the development of GVHD. Healthy individuals have a diverse intestinal bacterial flora, while the diversity of the intestinal flora significantly decreases during allo‐HSCT, the changes can be rapid and are thought to be due to the effects of antibiotics, intestinal inflammation and changes in diet.^26^ In this study, we also found many bacterial taxa were less abundant in the GVHD model mice. BBR could renovate the abundances of genus *Adlercreutzia*, *Dorea*, *Sutterella* and *Plesiomonas* and increase the abundance of *Lactobacillus* in GVHD mice. The abundance of these 5 kinds of genus was reported to be decreased in IBD or GVHD patient except *Plesiomonas* and re‐introduction of some of them could improve the disease in previous studies,^27^, ^28^, ^29^, ^30^, ^31^ which were mostly in consistent with our result.

Yet, it is believed that conditioning before allo‐HSCT and translocation of bacterial products in the recipients triggers the activation of NLRP3 inflammasome located in the cytoplasm in an inactive form. The basic cellular expression of NLRP3 inflammasome elements is regulated by a priming signal described as “signal 1”.^32^, ^33^ This signal is continuously delivered to the cells by LPS released from intestinal Gram‐negative bacteria after engaging TLR4 and its downstream effectors, including MyD88 and NF‐κB, which leads to the transcription of NLRP3 inflammasome components in an NF‐κb transcription factor‐dependent manner.^34^ In our study, we found that BBR can downregulate TLR4 signalling and remodel gut microbiota which NLRP3 inflammasome would not be primed by “signal 1”. In contrast to priming “signal 1”, functional activation of the synthesized NLRP3 inflammasome is mediated by “signal 2”, which is related to infection, cell activation, or cell/tissue damage.^35^, ^36^ Otherwise, we disclosed the dysfunction of colonic tight junction barrier and the mucosal damage induced by aGVHD, which mediates the functional activation of the synthesized NLRP3 inflammasome as “signal 2”. Tight junction proteins include Occludin, ZO and the claudin family, have a pivotal role in intestinal barrier function and translocation of the gut microbiota over the impaired intestinal barrier provokes inflammation.^37^, ^38^ The mucosal damage is the bystander effect of cytokines released in syngeneic implants.^39^ Animal studies have showed that the mortality of mice decontaminated the gut by antibiotics after BMT obviously decreased; the bystander effect was abrogated by the gut microbiota normalization.^40^ Protection of the mucosal barrier was also demonstrated to result in a lower incidence of GVHD.^41^ BBR was reported to protect the intestinal mucosal barrier,^42^ and might inhibit NLRP3 inflammasome by “signal 2”, which were also demonstrated in our results.

Through our work, we discovered that BBR significantly inhibited NLRP3 inflammasome activation and its inflammatory cytokines expression by gut microbiota remodelling and intestinal mucosal barrier protection, which involved in both “signal 1” and “signal 2” of NLRP3 inflammasome activation in aGVHD mice. However, there are some limitations of this study, for example, the mechanism needs to be shown and the gut microbiota change in GVHD mice after BBR treatment is still to be explored and clarified in future study.

## 5 CONCLUSION

Our results demonstrated that BBR significantly remodelled the gut microbiota to suppress TLR4 signalling and impaired gut barrier, which inhibited the vicious circle of NLRP3 activation and inflammatory cytokines release induced by GVHD. We have suggested a new molecular mechanism of BBR on GVHD protective through its inhibition of NLRP3 inflammasome primed and activated by “signal 1” and “signal 2”. Our finding indicated that BBR is a potential candidate for the GVHD treatment.

## CONFLICT OF INTEREST

The authors confirm that there are no conflicts of interest.

## AUTHOR CONTRIBUTIONS

**Yanna Zhao:** Methodology (equal); Project administration (equal). **Jiefeng Huang:** Methodology (equal). **Tianyi Li:** Data curation (equal). **Shuijuan Zhang:** Methodology (equal). **Chengping Wen:** Funding acquisition (equal); Supervision (equal). **Lipei Wang:** Funding acquisition (equal); writing – original draft (equal); writing – review and editing (equal).

## Data Availability

The raw data of Miseq sequences from 15 mice can be checked on NCBI Project under accession No. PRJNA723444 with NCBI SRA (Sequence Read Archive) under accession No. SRP1429931. All data utilized in this study are included in this article, and all data supporting the findings of this study are available on reasonable request from the corresponding author.
